# Potential of electro-sprayed purified mangiferin nanoparticles for anti-aging cosmetic applications

**DOI:** 10.1039/d3ra06308a

**Published:** 2023-12-01

**Authors:** Neungreuthai Chomchoei, Pimporn Leelapornpisid, Pratchaya Tipduangta, Padchanee Sangthong, Phakorn Papan, Busaban Sirithunyalug, Pawitrabhorn Samutrtai

**Affiliations:** a PhD Degree Program in Pharmacy, Faculty of Pharmacy, Chiang Mai University Chiang Mai 50200 Thailand; b Department of Pharmaceutical Sciences, Faculty of Pharmacy, Chiang Mai University Chiang Mai 50200 Thailand busaban.s@cmu.ac.th pawitrabhorn.s@cmu.ac.th; c Innovation Center for Holistic Health, Nutraceuticals and Cosmeceuticals, Faculty of Pharmacy, Chiang Mai University Chiang Mai 50200 Thailand; d Department of Chemistry, Faculty of Science, Chiang Mai University Chiang Mai 50200 Thailand

## Abstract

The fabrication of mangiferin nanoparticles using an electrospraying technique is a new and promising method for developing nanoparticles with higher efficiency and safety. This study aimed to fabricate mangiferin nanoparticles (MNPs) using cellulose acetate (CA) as a polymer at various parameters using electrospraying. Commercial mangiferin (CM) was purified from 88.46 to 95.71% by a recrystallization method to improve its purity and biological activities and remove any residue. The properties of recrystallized mangiferin (RM) were characterized using DSC, FTIR, X-ray diffraction (XRD) and HPLC. Then its biological activity and proteomics were determined. Proteomics analysis of RM showed that up-regulated proteins were involved in more biological processes than CM. MNPs were fabricated by varying the electrospraying parameters including voltage, the distance between the needle-tip–collector and flow rate. Skin permeation, release and irritation were also evaluated. The results revealed that the average particle size of the MNPs ranged between 295.47 ± 5.58 and 448.87 ± 3.00 nm, and had a smooth spherical morphology in SEM images. The MNPs also showed good potential in antioxidant and anti-aging properties. The encapsulation efficiency of MNPs was determined to be 85.31%. From skin permeation studies of CM, RM, and MNPs, the mangiferin content was found in the stratum corneum and dermis skin layers. Moreover, the MNPs solution had 23.68 ± 0.27% and 11.98 ± 0.13% of mangiferin in the stratum corneum and viable epidermis and dermis, respectively. Additionally, the irritation test by HET-CAM was mild and safe. Therefore, MNPs produced by electrospraying are a promising delivery system for cosmetic/cosmeceutical applications.

## Abstract 2

The fabrication of mangiferin nanoparticles using an electrospraying technique is a new and promising method for developing nanoparticles with higher efficiency and safety.

## Graphical abstract

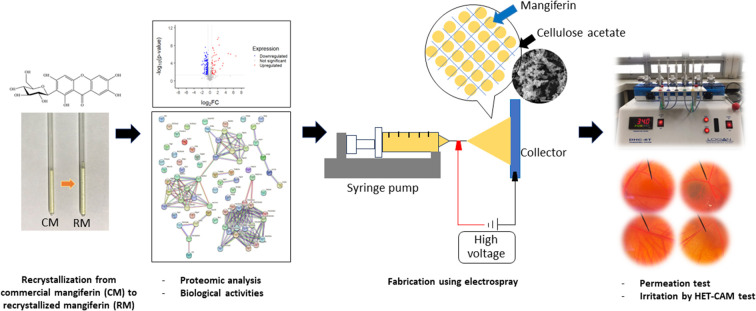

## 1 Introduction

**Fig. 1:**
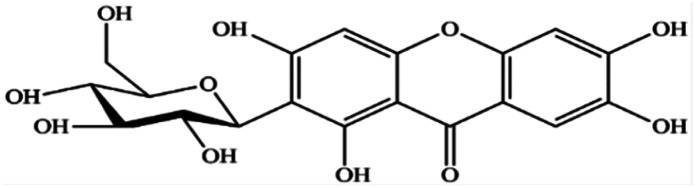
Chemical structure of mangiferin.

Mangiferin is a natural polyphenol with strong bioactivities and therapeutic properties (Fig. 1). It can be obtained from many plant species such as mango (*Mangifera indica* L.) and other herbal plants such as *Bombax malabaricum*, *Davallia solida* and *Phaleria macrocarpa* (Scheff.) *etc.* It can be found in several parts, especially in young mango leaves.^1,2^ Mangiferin has been reported to possess pharmacologic activities, including antioxidant, anti-inflammatory, antimicrobial, antiviral and anticancer and is used in the cosmetic industry. The chemical configuration of mangiferin influences its antioxidant activity.^3^ Mangiferin is a plant compound with a structure that includes a *C*-glycosyl linkage and polyhydroxy groups, which give it the ability to scavenge free radicals.^4^ It is as good or better at scavenging free radicals and reducing inflammation as other polyphenols, such as flavonoids and phenylpropanoic acids in DPPH assay.^5–9^ It is also more efficient at scavenging nitric oxide than curcumin.^10^ Mangiferin works by modulating the expression of pro-inflammatory genes in macrophages with NFκB pathway. It also inhibits the production of cytokines, such as GM-CSF, G-CSF, IL-6, TNF-α, TGF-β, and RANTES.^11,12^ Mangiferin also inhibits the release of arachidonic acid-derived inflammation mediators, such as PGE2 and LTB4, from activated macrophages. It does this by inhibiting the expression of COX-2 and nitric oxide synthesis by iNOS.^12,13^ Mangiferin has also been shown to be effective against bacteria and fungi. In one study, mangiferin inhibited the growth of seven strains of bacteria (*Bacillus pumilus*, *Bacillus cereus*, *Escherichia coli*, *Klebsiella pneumoniae*, *Staphylococcus aureus*, *Staphylococcus citreus* and *Salmonella agona*) and five species of fungi (*Aspergillus flavus*, *Aspergillus fumigatus*, *Saccharomyces cerevisiae*, *Trichoderma reesei* and *Thermoascus aurantiacus*).^7^ In addition to its anti-inflammatory and antioxidant properties, mangiferin also has potential for use in skincare products. For example, mangiferin derivatives have been shown to improve skin feel, moisturization, anti-aging, photoprotection, and antioxidant properties.^14^ One glycosylated derivative of mangiferin has been claimed to be useful in protecting skin from damage. Another patent claims that mangiferin or its derivatives can be used in cosmetic compositions to improve the structural quality of the skin or combat skin aging. Such compositions are claimed to be photoprotective, anti-collagenase, anti-elastase, anti-radical, and anti-tyrosinase.^15^ Mangiferin is often combined with other plant extracts, such as maple sap, lipophilic extract of sea buckthorn, *Eruca sativa* extract, and coffee extract, to deliver skin care benefits.^16^ Mangiferin is also an alpha-glucosidase inhibitor, which means it can inhibit the formation of advanced glycation end products in skin. There are harmful compounds that can contribute to skin aging.^17^

However, the poor solubility in water (0.16 mg mL^−1^), poor permeability and bioavailability have limited its pharmaceutical or cosmetic preparations.^18^ Different techniques are reported to improve the solubility and bioavailability of mangiferin, a natural compound with many potential health benefits. One way is to encapsulate mangiferin, which protects it from degradation and allows for controlled release at the target site.^19^ Encapsulating mangiferin with polysaccharides using the spray-drying method has been shown to be effective in improving its solubility and drug retaining ability.^20^ Another way to encapsulate mangiferin is to use the ionic gelation method with chitosan, a biocompatible polymer. This can help to overcome the limitations of mangiferin and make it more efficient for oral delivery. Mangiferin encapsulated using this method showed outstanding protection against induced oxidative stress in normal kidney epithelial cells.^21^ Recently, mangiferin-loaded carrageenan core–shell hydrogel beads have been developed to regulate the release of mangiferin for food protection.^22^

Nanotechnology is applied to fabricate novel materials and structures in the food, nutraceutical, pharmaceutical and cosmetic industries. Electrospraying or electrohydrodynamic atomization can be used to fabricate micro/nanoparticles of poorly water-soluble compounds.^23,24^ Electrospray mesoporous particles loaded with a novel chalcone (KAZ3, anticancer agent) showed a significantly improved solubility of poorly water-soluble drugs compared with the crystalline drug up to 30 fold. Additionally, *in vitro* and *ex vivo* studies of the electrospray mesoporous formulations showed potent permeability and cytocompatibility.^25^ Presently, many studies have focused on preparing and implementing mangiferin in the pharmaceutical industry. In contrast, only a few studies have shown the development of mangiferin in the cosmetic industry while it possesses antiaging, antioxidant, anti-inflammatory activities and photoprotection.^26^ For example, the complexation of mangiferin with β-cyclodextrin can be used to control the release of mangiferin, and improve their solubility, bioavailability, and protective effect on the membrane.^27,28^ In 2020, the treatment capacity of improvement with mangiferin-loaded transfersome vesicles showed the capacity to stimulate skin proliferation and repair damaged skin both *in vitro* and *in vivo*.^29^

Electrospraying or electrohydrodynamic atomization is a method for preparing micro/nano particles. The advantage of this method has been applied for several applications such as in the food industry, biotechnological sectors, pharmaceuticals and for cosmetic applications. Electrospraying is one of more interesting nanotechnology for fabricating uniform micro- and nanoparticles and enhancing its potential properties such as high encapsulation efficacy, prolonged release of drugs with hydrophobicity and low water solubility loading *etc.*^23^ The advantages of the versatile electrospraying technique is constituting a single-step, low energy and low cost material processing technology, which can deliver products having unique properties. In medicinal and pharmaceutical studies, electrospray particles have high biocompatibility, low toxicity and immunogenicity, and high solubility in many organic solvents (methanol, ethanol or dimethyl sulfoxide). The electrospraying setup consists of four major components a high voltage supplier, syringe pump, drug reservoir and conductive substrate.^23,24,30^

A recently published study reported that calcium-alginate microbeads were fabricated by electrospraying an aqueous alginate solution in distilled water containing calcium ions, which could be used as environmental-friendly cosmetic additives.^31^ Moreover, another study reported that the potential of the electrospraying technique indicates a viable route to enhance drug encapsulation and dissolution rates of a poorly water-soluble agents.^32^ The study aimed to focus on fabricating mangiferin nanoparticles as a modified active ingredient in cosmeceutical formulations using the electrospraying technique. Physicochemical properties were characterized and the biological activities evaluated. Moreover, the applicability to the biological process of proteomics were analyzed, and the characterization of the nanoparticles investigated. The permeation and release of mangiferin nanoparticles were also evaluated and any irritation was confirmed for cosmetic applications.

## 2 Results and discussion

### 2.1 Proteomics analysis

**Fig. 2:**
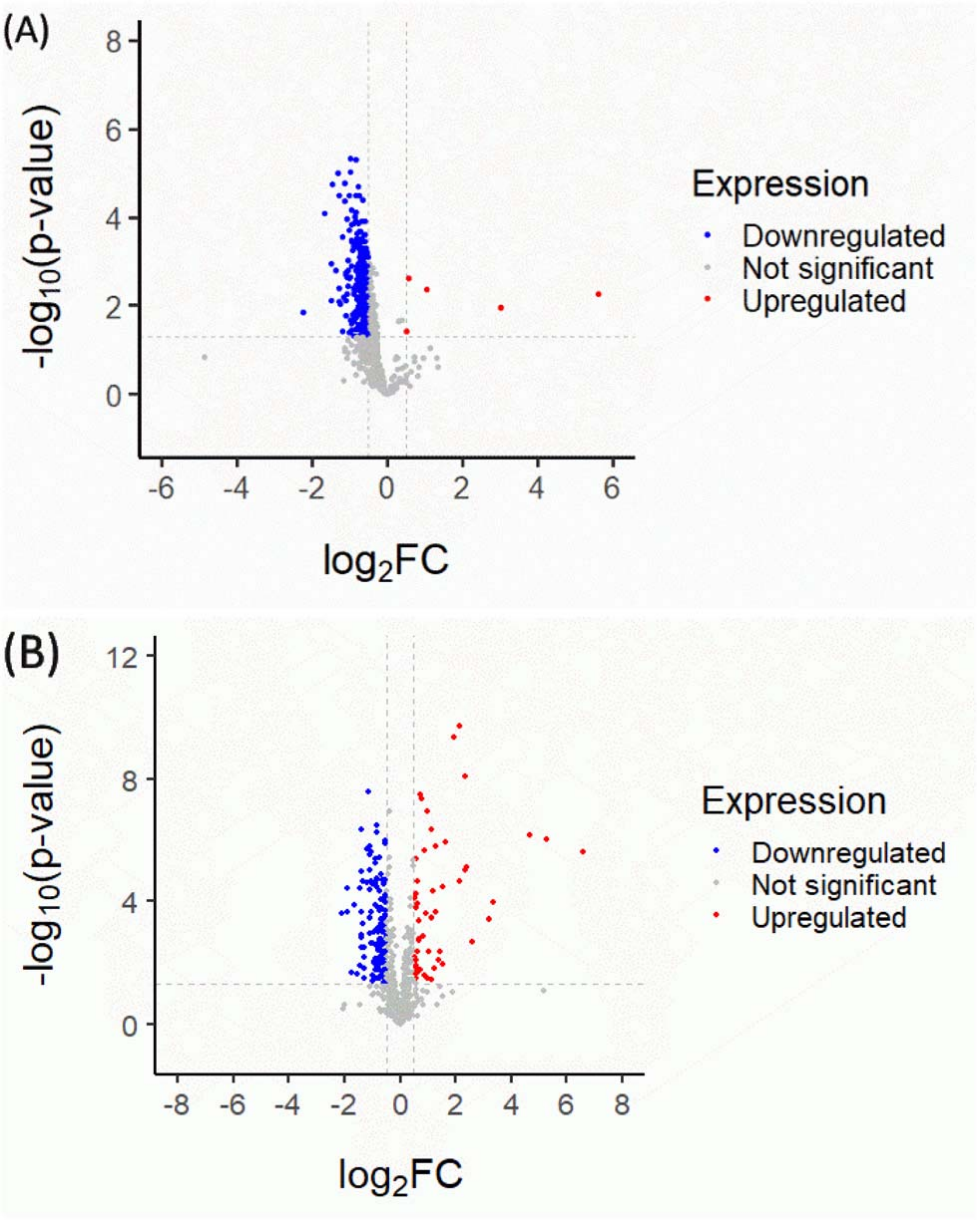
Volcano plots of CM (A) and RM (B). The plot shows a negative natural log of the *p*-value plotted against the base 2 logs of the change in each protein compared between CM and RM. Statistically significant results (*p* < 0.05) are plotted above the dashed line. Proteins significantly up- and down-regulated upon the CM and RM are shown as red and blue dots, respectively.

Quantitative proteomic analysis of CM and RM could potentially affect the anti-aging process in the *Mus musculus* fibroblast. Firstly, the normalized proteomics analysis showed different characteristics of the volcano plot of significantly changed protein expression in the CM and RM in Fig. 2. The global protein expression changes of CM were mostly down-regulated (37.28%; for 290 of 778 proteins). Whereas five significantly different proteins (*p* < 0.05 and proteins with ≥4 fold expression) were up-regulated (red region, 0.64%; for 5 of 778 proteins). The up-regulated proteins involved nestin (Q6P5H2 NEST_MOUSE), ATP-dependent RNA helicase DDX39A (Q8VDW0 DX39A_MOUSE), actin-related protein 2 (P61161 ARP2_MOUSE), Rho GDP-dissociation inhibitor 1 (Q99PT1 GDIR1_MOUSE) and treacle protein (O08784 TCOF_MOUSE). However, after the recrystallization process, the global protein expression change of RM was more up-regulated than that of CM (10.28%; for 80 of 778 proteins). The biological functions of the significantly different proteins of RM were considered related to the three up-regulated proteins of CM. The three up-regulated proteins were nestin (Q6P5H2 NEST_MOUSE), ATP-dependent RNA helicase DDX39A (Q8VDW0 DX39A_MOUSE) and actin-related protein 2 (P61161 ARP2_MOUSE), as shown in Table 1. Additionally, the down-regulated proteins of CM change to up-regulated proteins of RM comprised 60S ribosomal protein L21 (O09167 RL21_MOUSE), 26S proteasome non-ATPase regulatory subunit 14 (O35593 PSDE_MOUSE), 60S ribosomal protein L6 (P47911 RL6_MOUSE), protein disulfide-isomerase A6 (Q922R8 PDIA6_MOUSE) and dehydrogenase/reductase SDR family member 7B (Q99J47 DRS7B_MOUSE). The detailed biological process of these proteins is presented in Table 2.

**Table 1:** Description and functions of the significant proteins uniquely identified among those in the up-regulated CM. This information was obtained from the UniProtKB database

Accession	Protein name	Biological process
Q6P5H2 NEST_MOUSE	Nestin	Required for survival, renewal and mitogen-stimulated proliferation of neural progenitor cells
Q8VDW0 DX39A_MOUSE	ATP-dependent RNA helicase DDX39A	Involved in pre-mRNA splicing, required for the export of mRNA out of the nucleus
P61161 ARP2_MOUSE	Actin-related protein 2	Regulating gene transcription and repair of damaged DNA, the Arp2/3 complex promotes homologous recombination (HR) repair in response to DNA damage by promoting nuclear actin polymerization, leading to drive motility of double-strand breaks
Q99PT1 GDIR1_MOUSE	Rho GDP-dissociation inhibitor 1	Controls Rho proteins homeostasis. Regulating their stability and protecting them from degradation
O08784 TCOF_MOUSE	Treacle protein	Nucleolar protein acting as a regulator of RNA polymerase I by connecting RNA polymerase I with enzymes responsible for ribosomal processing and modification

**Table 2:** Description and functions of the significant proteins uniquely identified in the down-regulated proteins of CM change to up-regulated proteins of RM, this information was obtained from the UniProtKB database

Accession	Protein name	Biological process
O09167 RL21_MOUSE	60S ribosomal protein L21	Component of the large ribosomal subunit for the synthesis of proteins in the cell
O35593 PSDE_MOUSE	26S proteasome non-ATPase regulatory subunit 14	Maintains protein homeostasis by removing misfolded or damaged proteins, which could impair cellular functions; therefore, the proteasome participates in numerous cellular processes, including cell cycle progression, apoptosis, or DNA damage repair
P47911 RL6_MOUSE	60S ribosomal protein L6	Component of the large ribosomal subunit to synthesize proteins in the cell
Q922R8 PDIA6_MOUSE	Protein disulfide-isomerase A6	May function as a chaperone inhibiting aggregation of misfolded proteins, plays a role in platelet aggregation and activation by agonists such as convulxin, collagen and thrombin
Q99J47 DRS7B_MOUSE	Dehydrogenase/reductase SDR family member 7B	Putative oxidoreductase

**Fig. 3:**
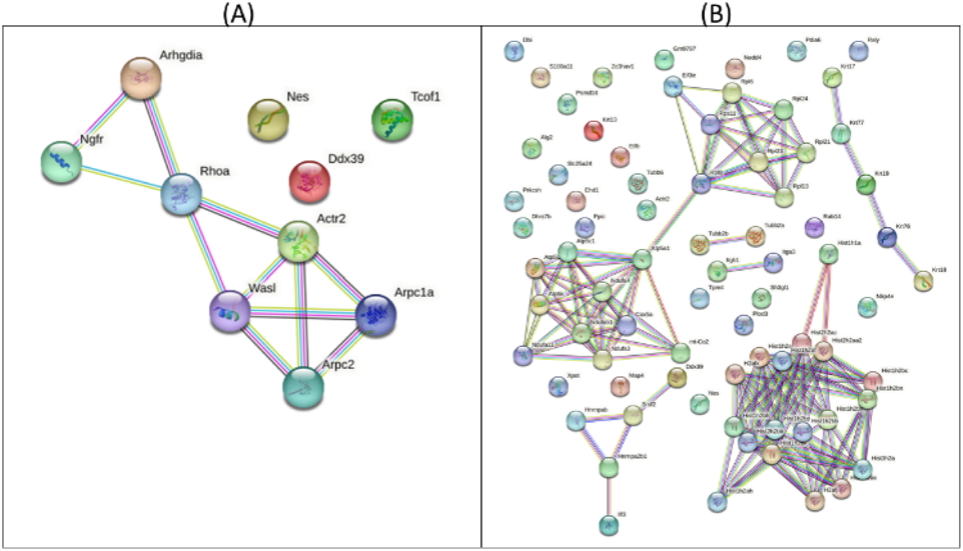
Protein–protein interaction map of >±4.01 fold proteins expressed in CM (A) and the RM (B).

The protein–protein interaction map, as illustrated in Fig. 3, indicated the >4.01 fold up-regulated proteins in CM and RM compared with those of the control. The top five potential pathways of the CM were actin nucleation (GO:0045010), positive regulation of actin filament polymerization (GO:0030838), positive regulation of cytoskeleton organization (GO:0051495), regulation of cytoskeleton organization (GO:0051493) and regulation of actin cytoskeleton organization (GO:0032956), as shown in Table 3 and Fig. 3A. Moreover, the top five potential pathways of RM were chromatin silencing (GO:0006342), organelle organization (GO:0006996), nucleosome assembly (GO:0006334), nucleosome organization (GO:0034728) and regulation of gene expression and epigenetics (GO:0040029), as shown in Table 4 and Fig. 3B. GO analysis of the ten up-regulated proteins in the CM group was mostly associated with the formation of an actin filament, the increase of actin polymerization and the regulation of cytoskeleton. In skin, the reduced assembly of the actin cytoskeleton leads to a decrease in collagen production.^33^ Moreover, RM was mostly associated with chromatin conformation, chromatin organization including the cellular level associated proteins, formation of the protein–DNA complex, RNA and gene expression. Other studies reported that changes in chromatin structure and function can contribute to the appearance of wrinkles and other signs of aging.^34^ The level of DNA methylation, a chemical modification of DNA that can affect gene expression, increases skin cells with age. This increase in DNA methylation can lead to the silencing of genes involved in skin repair and regeneration.^35^ However, the proteomic analyses of mangiferin have been limited to a few studies. One study showed that mangiferin may enhance the anti-oxidant abilities of cell repair and decrease cell damage by impacting the cell cycle. These results suggested that mangiferin improved the anti-aging activities of *Mus musculus* fibroblasts.

**Table 3:** Top 10 potential pathways of >+4.01 fold expressed proteins with unique peptide counts, identified in CM compared to control

Pathway ID	Description	False discovery rate (*p* < 0.05)
GO:0045010	Actin nucleation	2.99 × 10^−6^
GO:0030838	Positive regulation of actin filament polymerization	2.99 × 10^−6^
GO:0051495	Positive regulation of cytoskeleton organization	2.99 × 10^−6^
GO:0051493	Regulation of cytoskeleton organization	2.99 × 10^−6^
GO:0032956	Regulation of actin cytoskeleton organization	6.54 × 10^−6^
GO:0007015	Actin filament organization	3.82 × 10^−6^
GO:0034314	Arp2/3 complex-mediated actin nucleation	5.56 × 10^−5^
GO:0051130	Positive regulation of cellular component organization	0.00013
GO:0051128	Regulation of cellular component organization	0.00046
GO:0007266	Rho protein signal transduction	0.00096

**Table 4:** Top 10 potential pathways of >+4.01 fold expressed proteins with unique peptide counts, identified in RM compared to control

Pathway ID	Description	False discovery rate (*p* < 0.05)
GO:0006342	Chromatin silencing	9.75 × 10^−6^
GO:0006996	Organelle organization	1.27 × 10^−5^
GO:0006334	Nucleosome assembly	3.43 × 10^−5^
GO:0034728	Nucleosome organization	3.43 × 10^−5^
GO:0040029	Regulation of gene expression, epigenetic	3.43 × 10^−5^
GO:0071824	Protein–DNA complex subunit organization	3.43 × 10^−5^
GO:0016043	Cellular component organization	3.43 × 10^−5^
GO:0016458	Gene silencing	3.56 × 10^−5^
GO:0006333	Chromatin assembly or disassembly	3.56 × 10^−5^
GO:0006325	Chromatin organization	3.56 × 10^−5^

### 2.2 Physicochemical characterization of RM

#### 2.2.1 Differential scanning calorimetry (DSC)

**Fig. 4:**
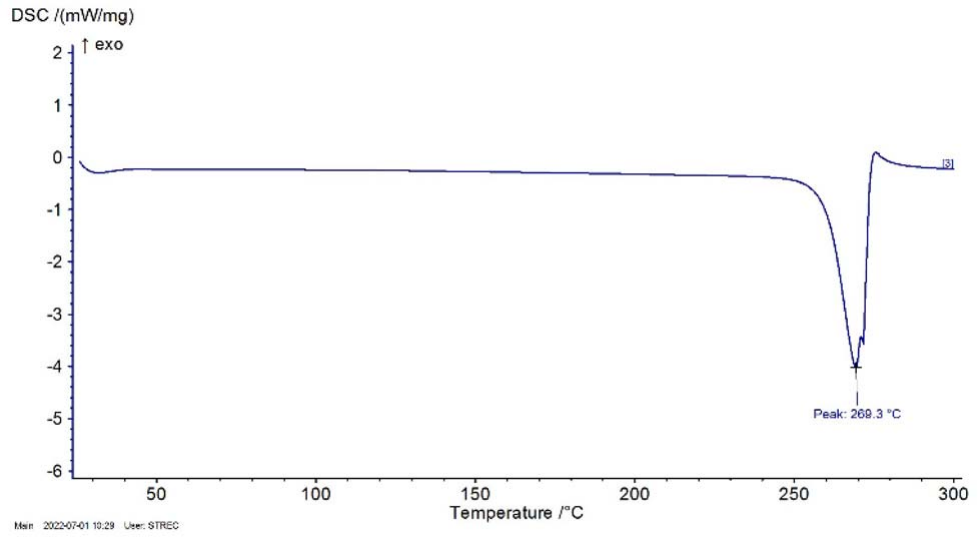
DSC profile of RM.

The DSC profiles are shown in Fig. 4. DSC curve of RM showed an endothermic peak at 269.3 °C due to the process of removing crystal water as expected. The results were compared with the DSC profile of Shiying, Y. *et al.*^36^ showing that mangiferin anhydrous indicated only one endothermic melting peak at 272 °C. The result of RM was nearly similar to the reference. However, the samples were proved to have a characteristic XRD pattern using XRD.

#### 2.2.2 Fourier transform infrared spectroscopy (FTIR)

**Fig. 5:**
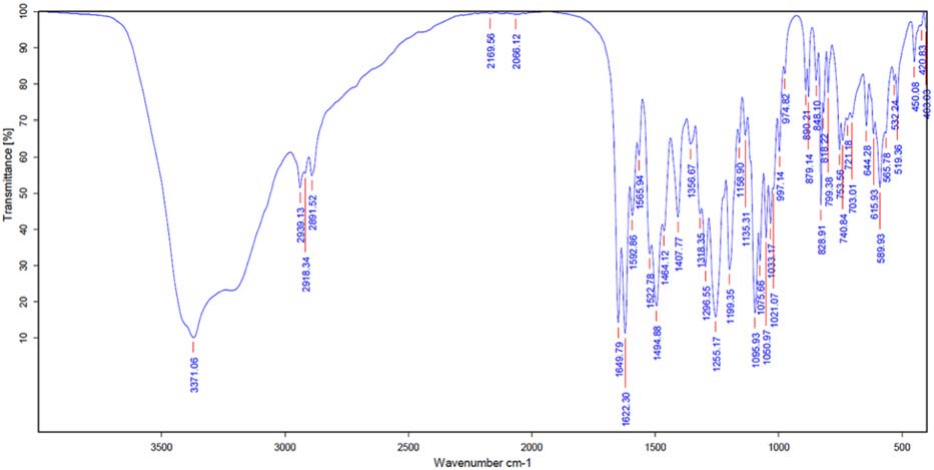
FTIR spectrum of RM.

FTIR spectrum results of RM showed peaks at 3371.06 cm^−1^ and indicated the presence of secondary OH^−^ bonds, 2918.34 cm^−1^ (C–H), 1649.79 cm^−1^ (C=O), 1622.30 cm^−1^ (C=C), 1494.88 cm^−1^ (CH–CH blending), 1255.17 cm^−1^ (C–O), 1199.35 cm^−1^ (C–O–C) and 1095.93 (O–H deformation). Particularly, the characteristic peak at 1021.07 cm^−1^ indicated the presence of C–C stretching in the mangiferin structure, as shown in Fig. 5 and Table 5. The results were compared with the FTIR analysis of mangiferin standard (MS)^37^ and pure mangiferin (PM).^38^

**Table 5:** FTIR peak values of RM and the study of Khurana, R. K. *et al.*, 2017 and Vo *et al.*, 2017 (wave number; cm^−1^)

Functional group (wave number; cm^−1^)	RM (cm^−1^)	MS (cm^−1^)^37^	PM (cm^−1^)^38^
O–H (3200–3400)	3371.06	3399	3397
C–H (2800–3000)	2918.34	2932.80	2902
C=O (1650–1800)	1649.79	1636.75	1649
C=C (1600–1670)	1622.30	—	1621
CH–CH (1450–1500)	1494.88	1497.10	—
C–O (1020–1275)	1255.17	1253	1256
C–O–C (1000–1300)	1199.35	—	1195
C–C (1020–1075)	1021.07	1023.22	—
O–H (deformation)	1095.93	—	1095

#### 2.2.3 X-ray powder diffraction (XRD)

**Fig. 6:**
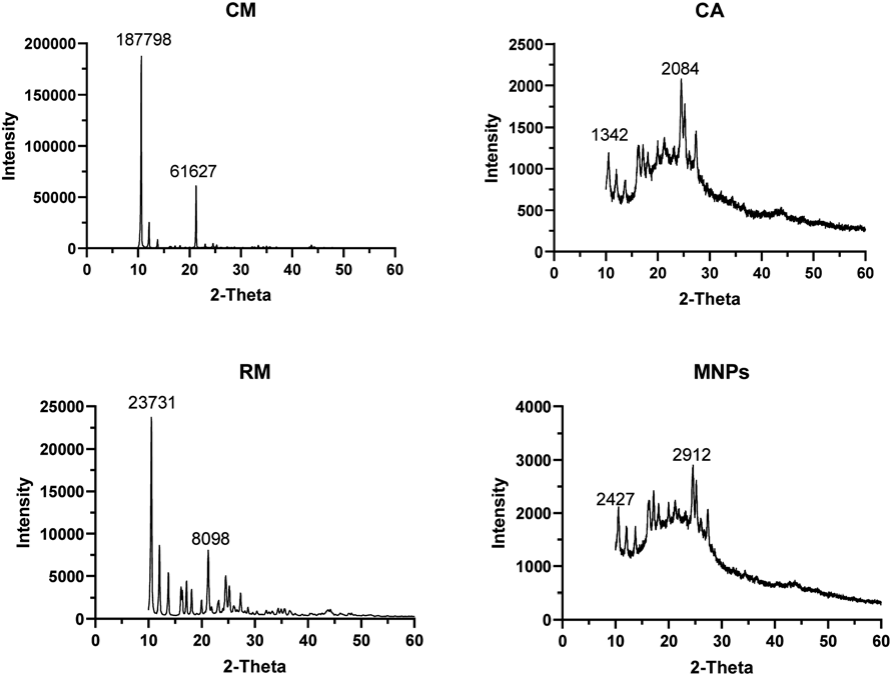
X-ray diffraction patterns of CM, RM, CA and MNPs.

The XRD patterns of CM, RM, CA and MNPs were illustrated in Fig. 6. CM and RM were in a crystalline form showing the characteristic peaks at 2*θ* of 10.61°, 21.29°, 33.40°, 43.75° and 51.57° for CM and at 10.55°, 21.22°, 33.36°, 43.72° and 51.55° for RM. Moreover, CA presented peaks at 24.55°, 30.24°, 43.76° and 51.19°, while MNPs showed peaks at 2*θ* of 24.57°, 30.26°, 43.78° and 50.58°. The XRD patterns of MNPs show amplified signals corresponding to RM between the characteristic peaks at 2*θ* 10 to 30 proving to be related to the formation described by other studies. Mangiferin has a broad absorbance peak around 10–30°.^38,39^ CM showed the intensity sharp peaks at 187 798 and 61 627 of 10.61° and 21.29°, respectively. RM indicates peak at 23 731 and 8098 of 10.55° and 21.22°, respectively. CA presented the prominent peaks at 1342 (19.99°) and 2084 (24.55°) whereas MNPs had prominent absorbance peaks at 2427 (17.17°) and 2912 (24.57°).

### 2.3 Chemical profile analysis: high performance liquid chromatography (HPLC)

**Fig. 7:**
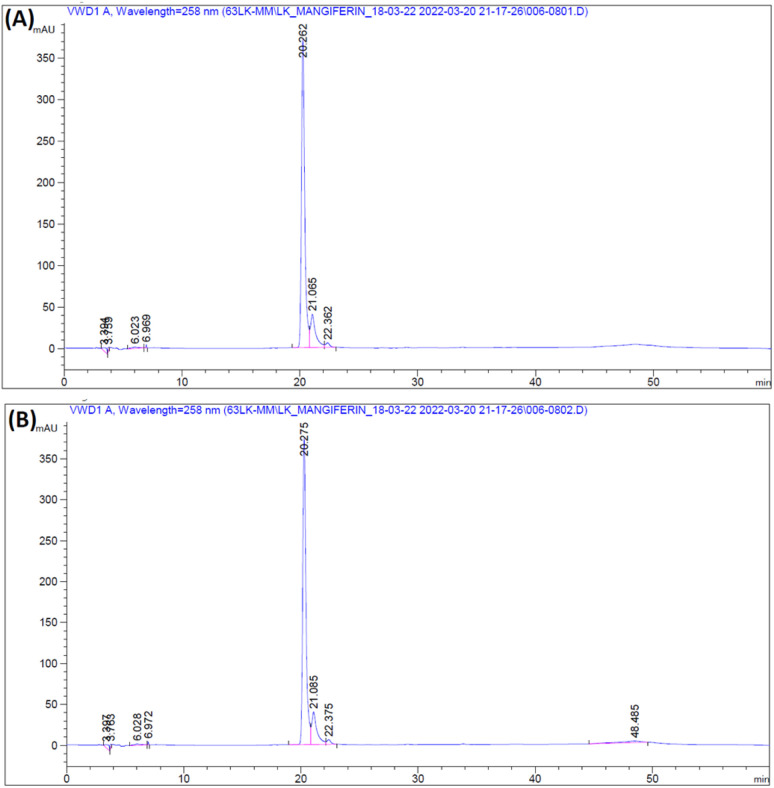
HPLC chromatogram of RM (A) and MS (B).

**Fig. 8:**
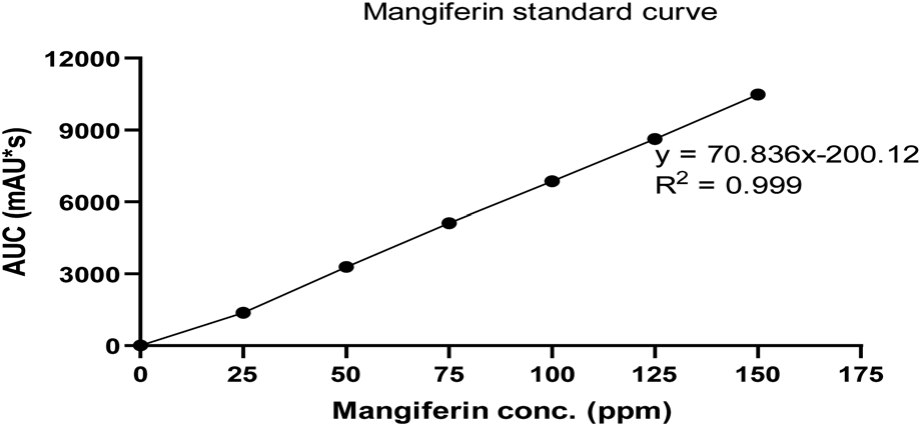
Linear calibration curve of MS in concentration of 0–150 ppm.

The RM solution in different concentrations was compared with the MS. The retention time of RM and MS were demonstrated at 20.262 and 20.275 min, respectively (Fig. 7). The purity of RM was 95.71%, from CM at 88.46%. After identifying the purified mangiferin, the qualitative as linearity, accuracy, precision, LOD and LOQ were evaluated. LOD and LOQ values were 11 μg mL^−1^ and 34 μg mL^−1^, respectively. Linear correlation was obtained between the peak area and concentration of three markers ranging from 25 to 150 μg mL^−1^. The values of the regression coefficients (*r*^2^) of the markers were higher than 0.99; thus, confirming the linearity of the methods (Fig. 8), and the high recovery values (96–106%) indicated a satisfactory accuracy. All R.S.D.s were below 3%.

### 2.4 Fabrication of mangiferin nanoparticles using electrospraying technique

**Fig. 9:**
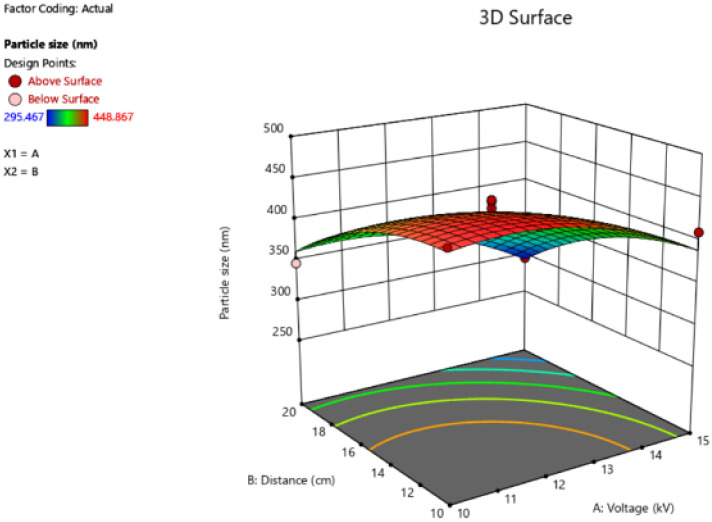
Response surface of the particle size, showing the interaction of the high voltage (kV) and the distance between needle-tip to the collector (cm).

CA is a water-insoluble polymer with excellent hydrophilicity, that can facilitate the diffusion of an aqueous solution. Another study reported that CA was used as a polymer for Ag-nanocubes embedded CA microspheres using the electrospray technique. The membranes have potential in quantitative investigations for surface-enhanced Raman scattering-based detection.^40^ In the study, the obtained particle size and polydispersity index (PDI) of MNPs indicate satisfaction (Table 6). The key of two variables on the output through 3D graphs are shown in Fig. 9. The high voltage and the distance between needle-tip to collector have an important effect by increasing high voltage and the distance resulting in a smaller particle size. The smallest average particle size was 295.47 ± 5.58 nm (run 11) which was produced by a high voltage of 15 kV and the distance between needle-tip to collector of 20 cm. On the other hand, the largest average particle size was 448.87 ± 3.00 nm (run 8) fabricated under 10 kV of high voltage and 10 cm of the distance between needle-tip to collector. Moreover, related reports revealed that the mixture of acetone and *N*,*N*-dimethylacetamide (DMAT) in the ratio of 2 : 1 v/v is a versatile solvent system for electrospinning CA nanofibers. CA/DMAT solution could slightly improve solution conductivity. Thus, these electrospray techniques conditions can be used to produce nanoparticles for delivery systems in cosmetic applications.^41^

**Table 6:** Average particle size and polydispersity index (PDI) of MNPs

Run	Particle size (nm)	PDI
1	426.07 ± 4.04	0.29 ± 0.02
2	435.87 ± 4.20	0.30 ± 0.01
3	346.47 ± 7.16	0.30 ± 0.01
4	400.40 ± 1.25	0.31 ± 0.01
5	309.63 ± 8.86	0.28 ± 0.01
6	409.03 ± 6.53	0.31 ± 0.04
7	418.87 ± 5.61	0.29 ± 0.01
8	448.87 ± 3.00	0.29 ± 0.01
9	434.17 ± 5.14	0.30 ± 0.01
10	323.60 ± 4.24	0.32 ± 0.03
11	295.47 ± 5.58	0.29 ± 0.01
12	422.50 ± 1.91	0.30 ± 0.01
13	410.10 ± 1.54	0.28 ± 0.01

### 2.5 Characterization and morphological of mangiferin nanoparticles

**Fig. 10:**
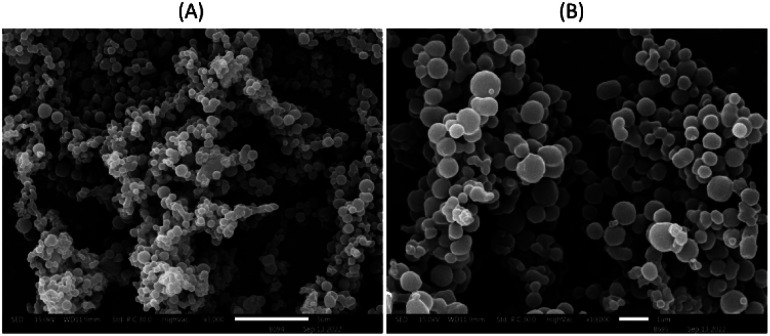
SEM image of MNPs using 2% cellulose acetate as a polymer loaded 2% recrystallized mangiferin at 5000× (A) and 10 000× (B).

The morphology and size distribution of MNPs are shown in Fig. 10. In the SEM images, MNPs exhibit spherical shapes and smooth surfaces with a tendency to result in aggregation in some locations, combining under high voltage. In general, the sizes of the MNPs in SEM images were related to those measured by the particle size analyzer. However, the MNPs with smaller or higher sizes could also be detected using SEM analysis.^42^ Therefore, MNPs of run 11 were selected for further study.

### 2.6 Biological activities

**Fig. 11:**
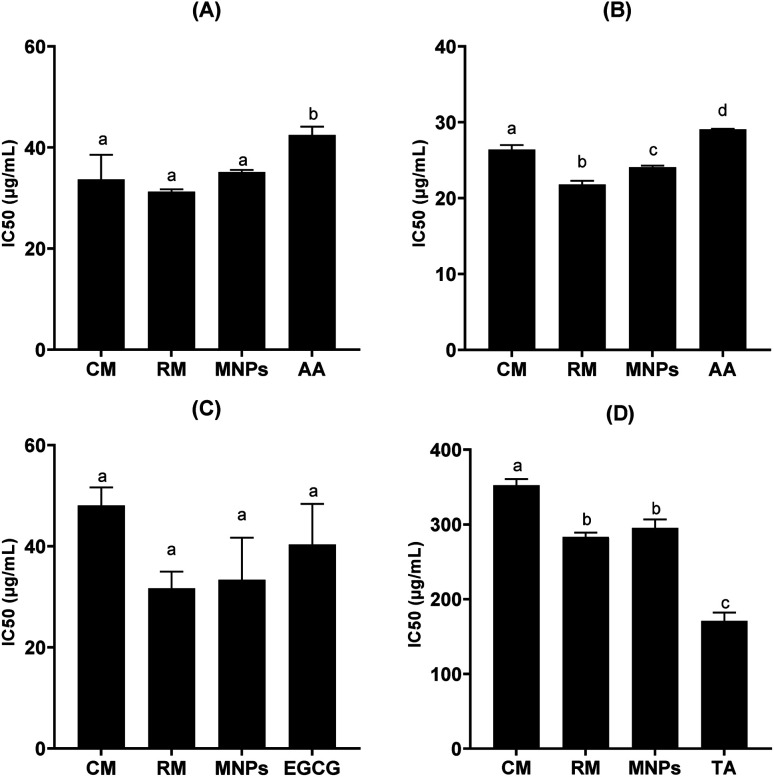
IC_50_ against DPPH free radical (A), IC_50_ against collagenase enzyme (B) IC_50_ against elastase enzyme (C) and IC_50_ against hyaluronidase enzyme (D) of commercial mangiferin (CM), recrystallized mangiferin (RM), mangiferin nanoparticles (MNPs), gallic acid (GC), l-ascorbic acid (AA), tannic acid (TA) and epigallocatechin gallate (EGCG), data are expressed as the mean ± SD (*n* = 3). a–d denote significant differences among samples in each experiment (*p* < 0.05).

Biological activities were inactivated by dose-dependence of mangiferin concentration, allowing us to calculate the concentrations of mangiferin required to scavenge 50% of the free radicals (IC_50_). All samples had significantly higher radical scavenging activity (RSA) than that of the positive control (*P* < 0.05). The ability of the CM, RM and MNPs to scavenge DPPH radicals compared with l-ascorbic acid is shown in Fig. 11A. The IC_50_ of CM was 33.68 ± 4.90 μg mL^−1^, whereas RM and MNPs were found to be 31.25 ± 0.47 μg mL^−1^ and 35.15 ± 0.41 μg mL^−1^, respectively. On the other hand, the positive control showed 42.48 ± 1.66 μg mL^−1^ in l-ascorbic acid. Therefore, mangiferin showed potent anti-oxidant activity that could be considered an active ingredient for skin aging.

Anti-aging activities of mangiferin were represented by collagenase inhibition, elastase inhibition and hyaluronidase inhibition are shown in Fig. 11B–D, respectively. Collagenase is the enzyme that breaks down the collagen peptide bonds, leading to skin aging.^43^ Interestedly, all samples had significantly higher collagenase inhibitory activity using FALGPA as the substrate compared with that of the positive control (*p* < 0.05). IC_50_ values against collagenase activity of CM, RM and MNPs were 26.41 ± 0.56 μg mL^−1^, 21.81 ± 0.47 μg mL^−1^ and 24.08 ± 0.10 μg mL^−1^, respectively (*p* < 0.05). l-Ascorbic acid showed the IC_50_ value of collagenase inhibitory activity at 29.05 ± 0.10 μg mL^−1^, as shown in Fig. 11B. To confirm the reversibility interaction between mangiferin and collagenase in a dose-dependent manner.^44^ Therefore, mangiferin had the potential for collagenase inhibition that could reduce the major characteristics of wrinkle formation.

Elastin is a composed principally of fibroblasts in an extracellular matrix of dermis layer for its elasticity and strength.^44^ It degrades by elastase enzyme. Fig. 11C illustrates that RM and MNPs showed excellent the IC_50_ value of elastase inhibitory activity at 31.36 ± 3.31 μg mL^−1^ and 33.36 ± 8.33 μg mL^−1^, respectively. EGCG, a positive control, leading to a 50% loss of anti-elastase was 40.35 ± 8.04 μg mL^−1^ IC_50_ of CM was 48.06 ± 3.57 μg mL^−1^. All samples had no significant different (*p* > 0.05). Thus, the result indicates that mangiferin could inhibit the degradation of extracellular matrix.

Hyaluronic acid is a key component of skin structure. The hydrolyzation of hyaluronic acid by hyaluronidase leads to loss of skin elasticity related to the skin aging process.^45^ In this study, all samples had significantly lower hyaluronidase inhibition than that of the positive control (*p* < 0.05). IC_50_ value of hyaluronidase inhibitory activities of CM, RM and MNPs were 352.55 ± 8.15 μg mL^−1^, 283.279 ± 5.67 μg mL^−1^ and 295.33 ± 11.19 μg mL^−1^, respectively. Tannic acid was used as a positive control, showing 171.02 ± 11.17 μg mL^−1^, as shown in Fig. 11D.

Mangiferin, a *C*-glycoside, is harder to break down into its aglycone and sugar parts than the more common *O*-glycosides. This is because the carbon–carbon bond between glucose and the phenolic compound is formed early in the formation of the benzophenone nucleus. As a result, the aglycone (norathyriol) is rarely found in many mangiferin-rich plants.^46,47^ Mangiferin is a stable compound, but it can react easily with oxidants. In solution, it has two ionizable functions (p*K*_a_, 7.5 and 12.2, respectively), and its UV absorption spectrum changes significantly with pH.^46^ Mangiferin is an efficient antioxidant that can neutralize natural reactive oxygen species (ROS), including hydroxyl radicals. Hydroxyl radicals can be produced by the Fenton reaction, which involves the use of transition metal ions (such as iron or copper), hydrogen peroxide, and a reducing agent (such as ascorbic acid). Mangiferin can act at different phases of the Fenton reaction to prevent or scavenge hydroxyl radicals. For example, it can chelate iron ions to prevent them from entering the reaction, or it can scavenge hydroxyl radicals directly. Mangiferin can also protect biomolecules from oxidative damage by breaking the chain of free radical propagation.^8,48–51^

Skin aging is linked to the breakdown of the skin's structure. Collagen, elastin, and hyaluronic acid are the main components of the skin's extracellular matrix (ECM). They are responsible for hydration, structural support, and keratinocyte proliferation. The breakdown of the ECM contributes to the formation of wrinkles.^44^ It has been shown that the ECM breaks down in the presence of reactive oxygen species (ROS).^52,53^ ROS produced in dermal fibroblasts and epidermal keratinocytes produce matrix metalloproteinases (MMPs), which break down collagen, leading to skin aging.^54^ In this study, mangiferin (CM, RM and MNPs) were found to show inhibitory effect of collagenase, elastase and hyaluronidase. Mangiferin is a natural compound that can donate electrons and interact with macromolecules. It has been shown to form complexes with macromolecules in the skin, as well as with enzymes such as collagenase and elastase. These enzymes are responsible for the breakdown of collagen and elastin, which are two important structural components of the skin.^55^ Moreover, mangiferin can inhibit the catalytic activity of collagenase and elastase, which means that it can slow down the process of skin aging. It can also scavenge free radicals, which are harmful molecules that contribute to oxidative stress, a major factor in skin aging.^44,56^ In addition to its anti-aging properties, mangiferin also has photoprotective activity. This means that it can protect the skin from the harmful effects of UV radiation. UV radiation is another major factor in skin aging.^57^

These results indicate that mangiferin (CM, RM and MNPs) may possess activity against skin aging through inhibiting collagenase, elastase and hyaluronidase. These could be used as an ingredient for anti-aging products in the cosmetic and cosmeceutical industry.

### 2.7 Encapsulation efficacy of mangiferin nanoparticles

The encapsulation efficacy of optimum MNPs was determined at 85.31%. High encapsulation efficiency in electrospray is this main advantage, making it attractive to many researchers.^58^ EE in this study was compared with other electrospray works. This value is higher than other methods to encapsulate proteins in a polymer. For instance, Yaghoobi *et al.*^59^ added streoptokinase solution to PLGA using electrospray and obtained an encapsulation efficiency of 90%. In contrast, the study of β-LG/mangiferin nanoparticles using nano-encapsulation technique showed a high EE% of 65%.^60^

### 2.8 *In vitro* release study

**Fig. 12:**
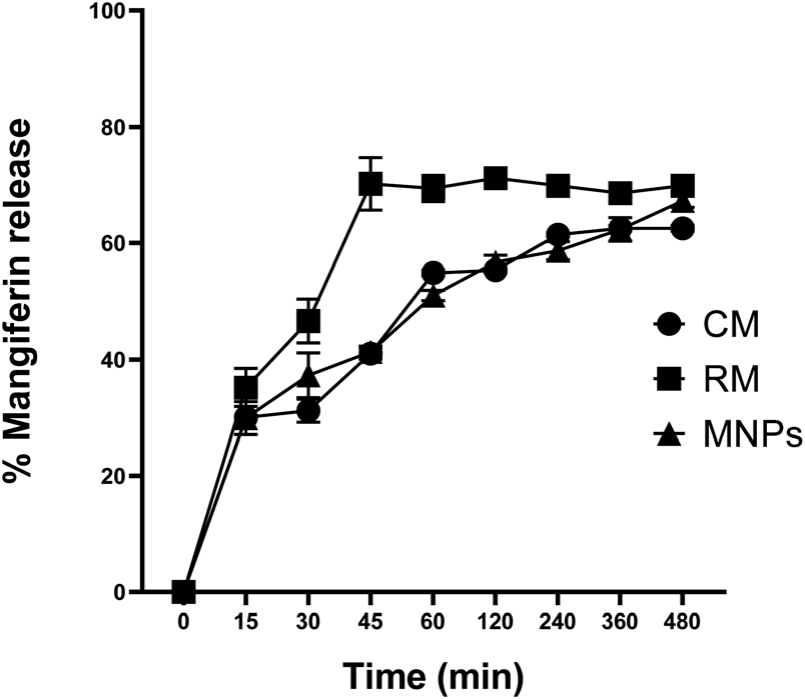
Mangiferin release profile of CM, RM and MNPs in PBS pH 7.4. The data are expressed as the means ± SD (*n* = 3).

The release profiles of CM, RM and MNPs are shown in Fig. 12. The results showed significant differences in the samples after 30 to 480 min. MNPs showed significant amounts of released mangiferin compared with that of CM at 30 to 480 min and RM (*p* < 0.05) at 60 to 480 min. CM and RM solutions exhibited a very fast release at the initial stage then constantly until 480 min, whereas the MNPs showed an extremely controlled release pattern. The results agreed with the related study of Samadarsi and Dutta.^61^ reporting that mangiferin release mechanisms from β-LG nanoparticles exhibited a non-Fickian diffusion. Other studies showed that it resulted from a polymer attributed mainly to the interactions between the present molecules (electrostatic interactions and hydrogen bonds) and the diffusivity of the nanoparticles.^60^ Hence, the study showed that CA was used as a polymer that might have affected the amount and rate of release of the drug from the nanoparticles.

### 2.9 *Ex vivo* permeation study

**Fig. 13:**
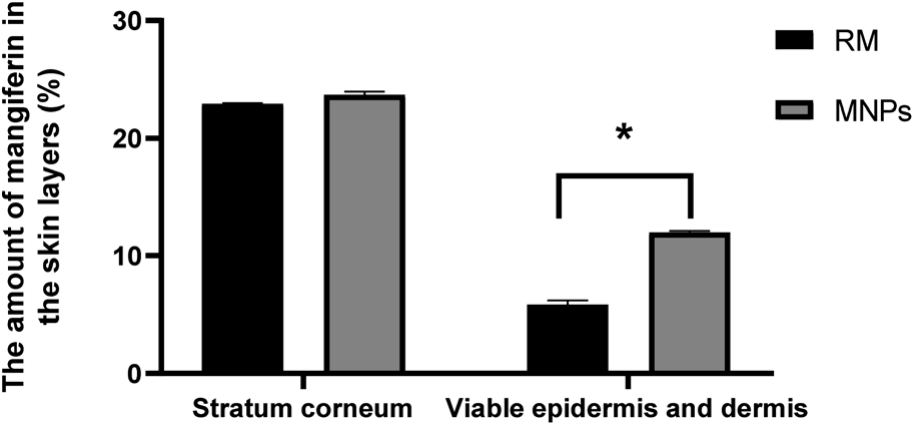
Amount of mangiferin in the skin layers applied as 0.2% RM solution and 0.2% MNPs solution the skin layers: the stratum corneum, viable epidermis and dermis (%). After 8 h of mangiferin application, the quantitative content of mangiferin in all the mangiferin in all the skin layers was measured using HPLC. Data are expressed as the mean ± SD (*n* = 3). * denotes significant differences among samples in each group (*p* < 0.05).

*Ex vivo* permeation of RM and MNPs were evaluated in terms of the amount of mangiferin in stratum corneum, viable epidermis and dermis and receiving solutions at 1, 2, 3, 4, 5, 6, 7 and 8 h. The skin permeation was studied using Franz-diffusion cells; the samples were input over newborn dorsal pig skin, as mentioned above that has been reported to represent similar structure and biochemical characteristics to human skin.^62^ Human skin experiments are limited and depend on international ethics considerations, so animal skin was used to replace human skin. Mangiferin possesses a branched glycoside structure demonstrating the ability to penetrate and pass through the skin (human skin, *ex vivo* study). The stratum corneum barrier did not stop the mangiferin. This might be associated with the fact that the mangiferin log *P* is between 1 and 3 (mangiferin log *P* = 2.73), corresponding to a weight less than 500 Da (422.34 Da).^44^ In this study, the amounts of mangiferin in the receiving chamber after 8 h remained undetected. On the other hand, the mangiferin concentration of both samples between the viable epidermis and dermis significantly differed (*p* < 0.05). The amount of mangiferin in the stratum corneum was insignificant, whereas MNPs presented a higher mangiferin content in the viable epidermis and dermis than in the RM solution. The MNPs solution revealed 23.68 ± 0.27 and 11.98 ± 0.13% of mangiferin in the stratum corneum, viable epidermis and dermis, respectively. RM exhibited 22.91 ± 0.06 and 5.84 ± 0.35%, respectively (Fig. 13). The results indicated that the amount of mangiferin permeated into skin layers from MNPs was higher than that of RM. The results corresponded with related studies reporting that CA, as a polymer in the formulation, improved active ingredient delivery, which has been used as semipermeable membranes of hydrophobic compounds.^41^ In addition, MNPs from the electrospraying technique can enhance the permeation rate, controlled release and prove effective in a poorly water-soluble substance.^5^

### 2.10 Irritation study by hen's egg chorioallantoic membrane (HET-CAM) assay

**Fig. 14:**
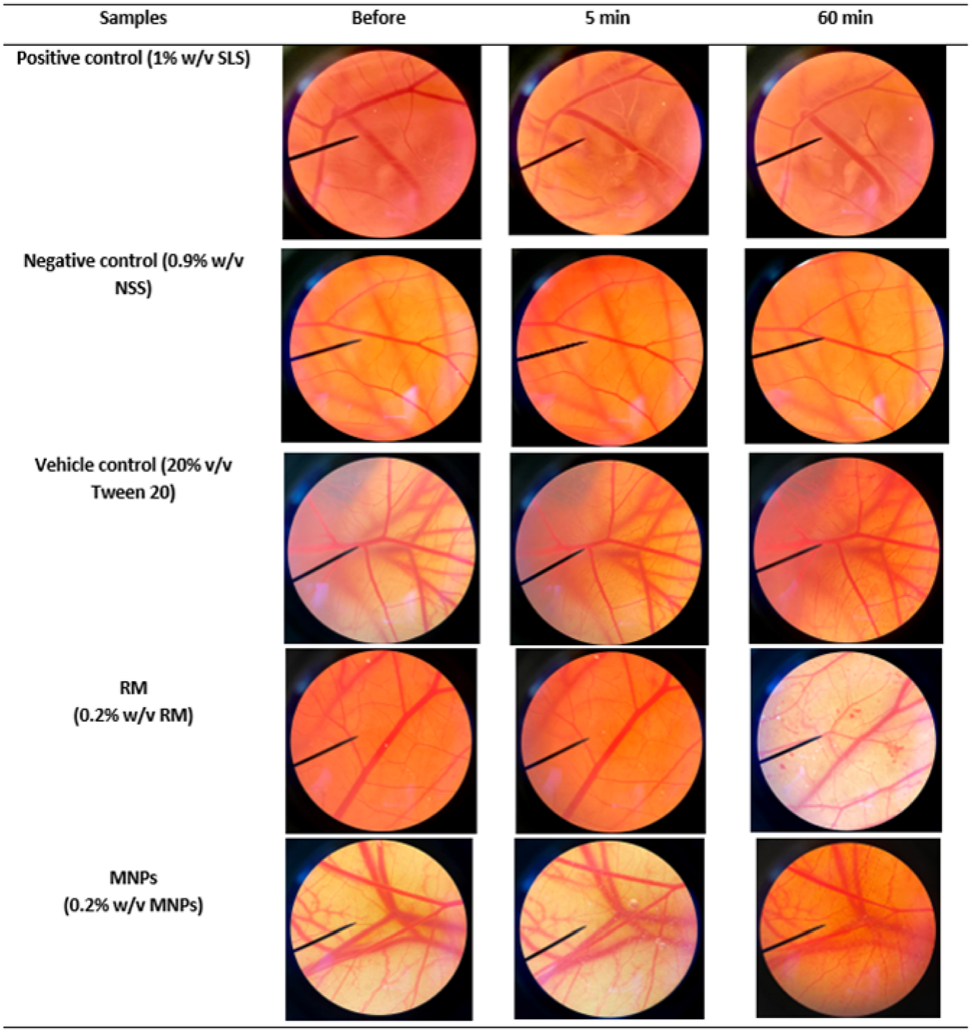
Photographs illustrating the effect of positive control (1% w/v SLS solution), negative control (0.9% w/v NSS), RM (0.2% w/v RM) and MNPs (0.2% w/v MNPs) on chorioallantoic membrane before exposing the sample (0 min), after 5 min and at the end of the experiment, 60 min.

Irritation is a major consideration when developing topical formulations. The HET-CAM test is a method using the chorioallantoic membrane (CAM) of fertilized eggs to assess the irritant effects of substances. The HET-CAM test is analogous to the Draize rabbit-eye test.^63^ In this study, RM and MNPs were dispersed in 20% (v/v) polysorbate. The results showed that the HET-CAM test was reliable in predicting irritation. A positive control of 1% sodium lauryl sulfate (1% w/v SLS) induced severe irritation with the irritation score (IS) of 12.40 ± 0.5. A negative control of normal saline solution (0.9% w/v NSS) induced no irritation with the IS of 0.0 ± 0.0. A vehicle control of 20% (v/v) polysorbate 20 also induced no irritation with an IS of 0.0 ± 0.0. Moreover, the SLS solution immediately irritated the CAM and caused hemorrhages. This was followed by coagulation and vascular lysis. All irritation signs were detected after 5 minutes of exposure and pronounced after 60 minutes. The SLS solution induced more pronounced vascular hemorrhages and vascular lysis after 60 minutes, as shown in Fig. 14. On the other hand, MNPs had less induced vascular haemorrhages and vascular lysis than RM because of the vehicle control (20% (v/v) polysorbate 20). Moreover, CA is biocompatible and could reduce the irritation making it suitable for skin use.^43^ However, all the samples did not indicate irritation on the CAM. Therefore, they were mild and safe nanoparticles for cosmetic applications.

## 3 Experimental

CM powder was obtained from Guangxi University of Chinese Medicine (purity 88.46%, lot number 20110530), MS (purity 99%), gallic acid (purity 98%), L929 cell line, CA (average *M*_n_ ∼ 30 000 by GPC, density 1.3 g mL^−1^ at 25 °C), epigallocatechin gallate, elastase from porcine pancreas and *N*-(methoxysuccinyl)-ala-ala-pro-val-4-nitroanilide were purchased from Sigma-Aldrich (St. Louis, MO, USA). l-Ascorbic acid 99.5% was procured from Loba Chemie PVT., Ltd. (Colaba, Mumbai, India). Isopropanol, glacial acetic acid, acetonitrile, dimethyl sulfoxide (DMSO), dimethylacetamide, sodium chloride, sodium dihydrogen phosphate and di-sodium hydrogen orthophosphate were purchased from RCL Labscan (Pathumwan, Bangkok, Thailand). GlutaMax™ (Gibco™) and dialysis tubing were purchased from Thermo Fisher Scientific (Waltham, MA, USA and Rockford, IL, USA, respectively). RapidGest SF surfactant was purchased from Waters (Waters Corporation, UK). Trypsin (sequencing grade modified trypsin) was purchased from Promega (Madison, WI, USA). Formic acid (LC-MS grade FA) was purchased from Sigma-Aldrich (Munich, Germany). Polysorbate 80 was purchased from Namsiang (Bangkok, Thailand). The stillborn piglet skin was obtained from Faculty of Medicine, Chiang Mai University, Thailand.

### 3.1 Preparation of RM

Firstly, CM (purity 88.46%) was purified by recrystallization according to the method described by Vo *et al.*^37^ and was recrystallized from a mixture of isopropanol and ultrapure water (1 : 1, v/v). Briefly, the mixture was boiled for 30 min while stirring. Then the hot solution was filtered and stored at 4 °C. The resulting precipitate was collected by filtration, washed with cold ethanol (three times) and dried in a vacuum oven at 80 °C for 3 h. The percentage of purity was calculated on the dried basis and was expected to be more than 90% after recrystallization.

### 3.2 Proteomics analysis

#### 3.2.1 Cell culture with the mangiferin and cells harvesting for LC-MS/MS

Mouse fibroblast cell lines (L929) were seeded in T-25 flasks with total volume of 5 mL at a density 1 × 10^5^ cells per mL in RPMI 1640 medium supplemented with 10% FBS and 1% penicillin/streptomycin. The cells were cultured in three conditions: CM and RM each at a concentration of 31.25 μg mL^−1^ for 24 hours. Then the cells were harvested by scraping them from the surface of the culture flask with sterile cold PBS. The cell suspension was then centrifuged at 14 000 rpm for 20 min at 4 °C. The supernatant was discarded, and the cell pellet was washed three times with sterile cold PBS. The cell pellet was then snap-frozen in liquid nitrogen and stored at −80 °C until proteomic analysis. For analysis, the cells were lysed in lysis buffer (0.5% SDS, 5 mM DTT in 0.1× PBS pH 7.4) with 1 mM PMSF.^64^

#### 3.2.2 Protein extraction and digestion

The cell lysate in lysis buffer (0.5% SDS, 5 mM DTT in 0.1× PBS pH 7.4) with 1 mM PMSF was prepared by sonication at 20 Hz for 5 s. Protein was purified by cold-acetone precipitation (1 : 5 (v/v)) and solubilized by 0.1% RapidGest SF in 20 mM ammonium bicarbonate. The concentration of protein was determined using Lowry's method. The amount of 3 μg protein was reduced (5 mM dithiothreitol at 72 °C for 30 min) and alkylated (15 mM iodoacetamide at RT in the dark for 1 h) before digestion using trypsin (sequencing grade modified trypsin; Promega, Madison, WI, USA) at a 1 : 20 trypsin/protein concentration ratio (37 °C, 4 h). The mixture was evaporated and resuspended with 0.1% formic acid in water.^64^

#### 3.2.3 Quantitative proteomics by LC/MS-MS analysis

The quantitative proteomics of CM and RM for protein expression profiles were investigated by tandem-mass spectroscopy using a NanoLC-system coupled with high resolution 6600 TripleTOF™ (AB-Sciex, Concord, ON, Canada). The LC conditions were as follows: mobile phase A and B were used, with mobile phase A composed of 0.1% formic acid in water and mobile phase B comprising 95% acetonitrile with 0.1% formic acid. The LC-method parameters comprised a 135 min long process for a single injection. The analytical column was maintained at 55 °C. In data-dependent acquisition mode of mass spectrometry, the MS scans over a mass range of 400 to 1600*m*/*z*, selecting the top 20 most abundant peptide ions with charge states in the range of 2 to 5 (positive mode) for fragmentation. This allows the identification of a wider range of peptides than would be possible if all ions within the mass range were fragmented. The dynamic exclusion duration was set at 15 s. The raw MS-spectra resulting (.wiff) file was extracted and annotated with protein sequences using the Paragon™ Algorithm by Protein Pilot™ software. The *Mus musculus* protein database, retrieved from UniProtKB (16 477 sequences). Protein thresholds (Unused Prot Score (Conf)) ≥ 0.05 with 1% false discovery rate (FDR) with ≥10 peptides/proteins were detected. The protein and peptide comparisons exhibiting >20% coefficient of variation (C.V.) between the replicates were rejected. The normalization of the relative protein abundances was performed using the R package. Volcano plot analysis using R Studio revealed a statistically significant increase in the expression (up-regulated) of each protein in the two experimental groups compared. Also, a statistically significant decrease in expression (down-regulated) was observed. Volcano plots were constructed using *q*-values and log_2_ FC values for all proteins between the two experimental groups. The proteins with a *q*-value less than 0.05 were the proteins that were statistically significantly expressed. Normalyzer DE,^65^ in which quantile-normalization was applied to expression data analysis after adding 1 to all expression values to avoid errors upon log transformation. The interpretation of the quantitative proteomics of CM and RM were set up at required confidence score of 0.7 (high confidence), which is the confidence value to be able to detect appropriate interaction data. Proteins with ≥4 fold expression compared with the control were further used for protein–protein interaction mapping using the STRING Database, Ver 12.0 (https://string-db.org/). Then the up-regulated CM and RM were selected to identify significantly different proteins and considering the biological functions using UniProt^KB^ (https://www.uniprot.org/).

### 3.3 Physicochemical characterization of RM

#### 3.3.1 Differential scanning calorimetry (DSC)

DSC is a useful technique to investigate physicochemical properties, especially melting point, of CM and RM. The sample was placed in a standard aluminum pan and cover was measured at a heating of 25 to 300 °C temperature range at a constant heating rate 10 °C min^−1^ in a dynamic nitrogen atmosphere (flow rate = 30 mL min^−1^) using DSC (NETZSCH DSC 204 F1 Phoenix®, NETZSCH-Gerätebau GmbH, Germany).^27^

#### 3.3.2 Fourier transform infrared spectroscopy (FTIR)

The functional group of mangiferin and its interaction was obtained using the FITR technique. The FTIR profile was executed for RM. The KBr disk was scanned at a resolution of 4.0 cm^−1^ over a wave of the number region of 4000 to 400 cm^−1^, and the number of scans as 64 using FTIR spectroscopy (Bruker, INVENIO®S). A blank KBr disc was used as a background.^27^

#### 3.3.3 X-ray diffraction (XRD)

XRD of the CM, RM, CA and MNPs was performed using an automated diffraction system (RIGAKU, Model SMARTLAB). Measurements were carried out under exposure of Cu Kα radiation of wavelength 1.5405 Å, at 40 kV, 30 mA. The speed was 10 dpm, step 0.01 min^−1^, over the range of 2*θ* = 10 to 60°.^27^

### 3.4 Chemical profile analysis: high performance liquid chromatography (HPLC)

MS, CM and RM, dissolved in a mixture of isopropanol and ultrapure water (1 : 1, v/v), were analyzed using HPLC according to the method described by Schieber *et al.*^66^ with some modifications. Briefly, the HPLC system using a C18 analytical column (5 μm, 4.6 × 250 mm) was used with the detection wavelength of 258 nm (Hewlett Packard: Agilent HP1100, USA). The mobile phase of HPLC consisted of 2% acetic acid in water (A) and acetonitrile: 0.5% acetic acid (1 : 1, v/v) (B). The gradient elution was performed as follows: 0 to 2 min, 5% of B; 2 to 10 min, 5 to 25% of B; 10 to 40 min, 25 to 55% of B; 40 to 45 min, 55 to 90% of B; 45 to 50 min, 90 to 55% of B. The flow rate was maintained at 0.8 mL min^−1^, the injection volume at 20 μL, and the column temperature kept at 25 °C.

### 3.5 Fabrication of mangiferin nanoparticles using electrospraying technique

Mangiferin nanoparticles were fabricated using electrospraying based on the method described by Nguyen *et al.*^67^ with some modifications. A nontoxic, biodegradable polymer, CA, was used as a polymer to fabricate mangiferin nanoparticles (MNPs). MNPs were optimized using the design of experiments (DOE). The precursor solution for electrospraying was prepared using ratios of RM : CA, namely, 1 : 1, at a total solution concentration of 4% (w/v). The precursor solution was prepared by co-dissolving the RM and polymer in a mixture of isopropanol : acetone : dimethylacetamide (3 : 3 : 4, v/v/v). The optimum electrospraying conditions were a high voltage of 10 to 15 kV, the distance between needle tip to the collector of 10 to 20 cm and the flow rate was fixed at 0.3 mL h^−1^. Table 7 shows the experimental condition generated from the Design-Expert 13 software base on the central composite (CCD) of the electrospray conditions for the preparation.

**Table 7:** Experimental conditions generated from the Design-Expert 13 software based on the central composite (CCD)

Run	Factor 1 A: distance (cm)	Factor 2 B: high voltage (kV)
1	12.5	15
2	12.5	15
3	10	20
4	12.5	15
5	16.04	15
6	12.5	15
7	8.96	15
8	10	10
9	12.5	15
10	12.5	22.07
11	15	20
12	12.5	7.93
13	15	10

### 3.6 Characterization of mangiferin nanoparticles

#### 3.6.1 Size and size distribution

The particle size and polydispersity index (PDI) of MNPs were determined in 20% polysorbate 80 in DI water using a dynamic light scattering particle size analyzer (Malvern®, Zetasizer ZS).^39^

#### 3.6.2 Scanning electron microscope (SEM) analysis

The SEM studies were performed to visualize the surface morphology of MNPs according to the method described by Razura *et al.*^39^ MNPs were mounted on aluminum stubs and sputter coated with a thin layer of gold at 10 torr vacuum before examining using an SEM (JEOL®, JSM-IT300). Both low and high-magnification images were obtained to confirm the particle sizes and interpreted by ImageJ software.

### 3.7 Biological activities

#### 3.7.1 DPPH radical scavenging activity

The anti-oxidant activities of CM, RM and MNPs at the various concentrations were measured regarding their RSA. The DPPH scavenging activity was performed as described by Nanjo F. *et al.*^68^ Briefly, the samples were mixed with 166 μM DPPH in absolute ethanol and incubated at room temperature without light for 30 min. Absorbance was measured at 520 nm using a microplate reader (BMG Labtech, SPECTROstar Nano). l-Ascorbic acid were used as a positive control. The percentage inhibition was calculated using the equation below.1
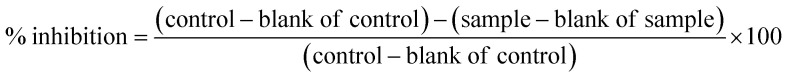


#### 3.7.2 Anti-collagenase assay

The anti-collagenase activity was evaluated based on the method described by Wang *et al.*^43^ with some modifications. Briefly, the reagent was prepared as follows: 50 mM tricine buffer (pH 7.5), 0.8 mM, FALGPA (*N*-[3-(2-furyl)acryloyl]-leu-gly-pro-ala) and 0.1 units of collagenase. Then CM, RM and MNPs at various concentrations were added to the test tube and incubated at room temperature for 15 minutes. FALGPA was transferred to the solution and incubated at room temperature. l-Ascorbic acid were used as a positive control, and absorbance was measured at 335 nm and 513 nm for 20 min using a microplate reader (Molecular Devices, SpectraMax M3). The percentage inhibition was calculated using eqn (1).

#### 3.7.3 Anti-elastase assay

The anti-elastase activity was evaluated based on the method described by Kim *et al.*^69^ with some modifications. CM, RM and MNPs at various concentrations were added to the 96-well plates. Then, it was mixed with 0.1 mol per L Tris–HCl buffer (pH 7.5) and 10 mM *N*-(methoxysuccinyl)-ala-ala-pro-val-4-nitroanilide (MAAPV). The plates were incubated at room temperature for 15 minutes. Elastase (0.3 units per mL) was added and incubated at room temperature for 15 minutes. Epigallocatechin gallate was used as a positive control, and absorbance was measured at 410 nm using a microplate reader (BMG Labtech, SPECTROstar Nano). The percentage inhibition was calculated using eqn (1).

#### 3.7.4 Anti-hyaluronidase assay

Antihyaluronidase activity was determined based on the turbidimetric method described by Chaiyana *et al.*,^45^ with some modifications. The hyaluronidase inhibitory activity of CM, RM and MNPs at various concentrations were investigated using a spectrophotometric assay. Firstly, 15 units per mL of hyaluronidase solution was prepared by dissolving hyaluronidase from bovine testes in 20 mM phosphate buffer at pH 5.35 containing 0.01% w/v BSA and 77 mM NaCl. Subsequently, hyaluronidase solution was added to the sample in a micro centrifuge tube and incubated at 37 °C for 10 min. Secondly, 0.03% w/v hyaluronic acid in phosphate buffer at pH 5.35 was added, and further incubated at 37 °C for 45 min. Subsequently, acidic albumin solution was added and incubated at room temperature for 10 min. Then the mixed solution was added to a flat-bottomed 96-well plate. Finally, the absorbance was measured at 600 nm using a microplate reader (BMG Labtech, SPECTROstar Nano). l-Ascorbic acid and gallic acid were used as a positive control, and the percentage inhibition was calculated using eqn (1).

### 3.8 Encapsulation efficacy of mangiferin nanoparticles

The encapsulation efficacy (EE%) of MNPs was determined according to the indirect method described by Nguyen *et al.*^67^ with some modifications. Briefly, the sample collected after electrospraying was analyzed to quantity the encapsulated mangiferin. MNPs were dissolved in 50% (v/v) IPA in ultrapure water. Then one milliliter of the supernatant of MNPs was collected after being centrifuged at 10 000 rpm for 40 min (Sorvall ST16R, Thermo Fisher Scientific, Germany). The supernatant was filtered through a PTFE membrane with 0.22 μm pore size. Afterward, the amount of encapsulated mangiferin and free mangiferin dissolved in 50% (v/v) IPA in ultrapure water was determined using HPLC analysis. The EE% was calculated using the equation below.2
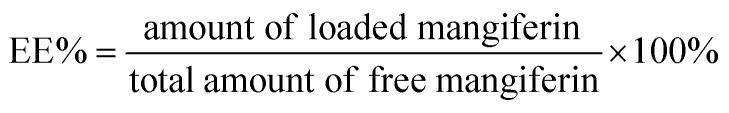


### 3.9 *In vitro* dialysis release study

The mangiferin release from the nanoparticles using dialysis bag diffusion was performed according to Kuo and Chung^70^ with some modifications. Briefly, 2 mL of CM, RM and MNPs were dispersed in 5 mL of PBS buffer at pH 7.4 and stored in the dialysis bags (molecular weight cut off 10 kDa, 35 mm dry I.D.). The dialysis bag was maintained at 32 ± 0.5 °C by stirring the medium and collecting at 0, 15, 30, 45, 60, 120, 180, 240, 360 and 480 min. The sample was collected at various times, and the mangiferin content was analyzed using HPLC at 258 nm.

### 3.10 *Ex vivo* permeation study

The skin permeability of RM dispersion and the mangiferin nanoparticles dispersion were determined using a Franz-diffusion cell. The study was performed according to Ochocka *et al.*^44^ with some modifications. Stillborn dorsal piglet skin was used in the experiment due to its ease of acquisition and ethical exemption. These readily available skin models can serve as suitable replacements for human skin *in vivo*, facilitating the evaluation of topical product bioequivalence.^71^ Dorsal pig skin was excised, and the fat was removed and placed on the receptor chamber. The samples were added to the donor chamber. The medium condition was performed at 32 ± 0.5 °C under stirring. One millilitre of the medium was collected each 1 h until 8 h and was replaced with fresh medium. After 8 hours, the penetrants were removed from the skin, and the stratum corneum was separated by tape-stripping method using adhesive tape (20 fragments). HPLC evaluated mangiferin content in the receiving chamber. The tapes and all skin layers were separately extracted with 50% isopropyl alcohol in DI water, and then the mangiferin content was evaluated using HPLC.

### 3.11 Irritation study by hen's egg chorioallantoic membrane (HET-CAM) assay

The irritation of RM and MNPs were investigated using the HET-CAM assay according to Steiling *et al.*^72^ and Somwongin *et al.*^73^ with some modifications. The cosmetic formulation was applied in different modes (short term and long term) depending on its application. Cleaning and rinsing products lead to acute irritation in the short term application mode, whereas skin care (cream or gel) causes allergic or chronic wounds in the long term. Irritation signs occurring on CAM were observed for 5 min (short term application) and after 60 min (long term application).^27^ Briefly, the fertilized hen's egg were incubated for 7 days at 37 ± 0.5 °C and humidity of 55 ± 7% RH. The eggshell above the air chamber was opened by rotating the cutting blade. Then the inner membrane was carefully removed using forceps to avoid vessel bleeding. The samples were exposed to 30 μL of (10 mg mL^−1^) CAM. Sodium lauryl sulphate (1% w/v) and normal saline solution (0.9% w/v NaCl) were used as a positive and negative control, respectively. The irritation signs including hemorrhage, vascular lysis and coagulation on CAM were observed under the stereomicroscope at 5 minutes. The irritation index score (IS) was calculated using the equation below:3
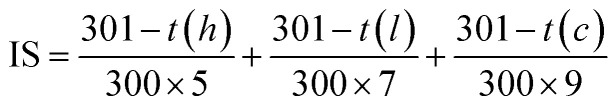
where *t*(*h*) is the first time vascular hemorrhage occurred, *t*(*l*) is the first time vascular lysis occurred and *t*(*c*) is the first time vascular coagulation occurred. Then IS was classified as follows: 0.0 to 0.9 is no irritation, 1.0 to 4.9 is slight irritation, 5.0 to 8.9 is moderate irritation, and 9.0 to 21.0 is severe irritation. The irritation signs on CAM were observed again after 60 min to determine the long term irritation. The experiments were independently performed in triplicate.

### 3.12 Statistical analysis

All the statistical analysis was performed using SPSS Program, Version 17.0 and *p* < 0.05 was considered the minimum level of significance in all cases. The results are expressed as mean with standard deviation (SD). The parametric data were evaluated using the *t*-test or one-way ANOVA with *post hoc* Turkey's test.

## 4 Conclusions

Recrystallization could improve the purity and biological activity of CM. The physicochemical and chemical property of the RM were illustrated by the characteristic of mangiferin. Proteomic analysis results suggest that RM can be used as an anti-aging agent. The electrospraying technique is one of the potential methods for preparing MNPs. Fabricating MNPs using CA as a polymer was effective and enhanced its properties. High voltage and the distance between the needle tip and collector were found to be significant parameters in determining the size of the MNPs. The optimum parameters resulted in MNPs with the smallest size of 295.47 ± 5.58 nm, spherical shape, high encapsulation efficacy of up to 85.31% and good permeation and release. The MNPs presented a higher mangiferin content in the viable epidermis and dermis than in the RM significantly differed. Biological activities indicate that MNPs had significantly higher radical scavenging activity and anti-collagenase activity than l-ascorbic acid as a positive control, as well as no irritation in the HET-CAM test. Our study showed that MNPs are a novel and promising ingredient for cosmetic and cosmeceutical applications.

## Author contributions

N. C. designed the experiments, analyzed the data and wrote the manuscript. P. S. and P. P. performed the proteomic analysis. All authors discussed the results and commented on the manuscript.

## Conflicts of interest

The publication has been approved by all co-authors and the responsible authorities at the institute(s) where the work has been carried out.

## Supplementary Material
